# Municipal policies and plans of action aiming to promote physical activity and healthy eating habits among schoolchildren in Stockholm, Sweden: a cross-sectional study

**DOI:** 10.1186/1748-5908-4-47

**Published:** 2009-08-03

**Authors:** Karin Guldbrandsson, Karin Modig Wennerstad, Finn Rasmussen

**Affiliations:** 1 Division of Social Medicine, Department of Public Health Sciences, Norrbacka floor 5, Karolinska Institutet, SE-171 76 Stockholm, Sweden; 2 The Swedish National Institute of Public Health, Östersund, Sweden; 3 Child and Adolescent Public Health Epidemiology Unit, Department of Public Health Sciences, Norrbacka floor 5, Karolinska Institutet, SE-171 76 Stockholm, Sweden

## Abstract

**Background:**

Promoting physical activity and healthy eating habits by structural measures that reach most children in a society is presumably the most sustainable way of preventing development of overweight and obesity in childhood. The main purpose of the present study was to analyse whether policies and plans of action at the central level in municipalities increased the number of measures that aim to promote physical activity and healthy eating habits among schoolchildren aged six to 16. Another purpose was to analyse whether demographic and socio-economic characteristics were associated with the level of such measures.

**Methods:**

Questionnaires were used to collect data from 25 municipalities and 18 town districts in Stockholm County, Sweden. The questions were developed to capture municipal structural work and factors facilitating physical activity and the development of healthy eating habits for children. Local policy documents and plans of action were gathered. Information regarding municipal demographic and socio-economic characteristics was collected from public statistics.

**Results:**

Policy documents and plans of action in municipalities and town districts did not seem to influence the number of measures aiming to promote physical activity and healthy eating habits among schoolchildren in Stockholm County. Municipal demographic and socio-economic characteristics were, however, shown to influence the number of measures. In town districts with a high total population size, and in municipalities and town districts with a high proportion of adults with more than 12 years of education, a higher level of health-promoting measures was found. In municipalities with a high annual population growth, the number of measures was lower than in municipalities with a lower annual population growth. Another key finding was the lack of agreement between what was reported in the questionnaires regarding existence and contents of local policies and plans of action and what was actually found when these documents were scrutinized.

**Conclusion:**

Policy documents and plans of action aiming to promote physical activity and healthy eating habits among schoolchildren aged six to 16 in municipalities and town districts in Stockholm County did not seem to have an impact on the local level of measures. Demographic and socio-economic characteristics of the municipalities and town districts were on the other hand associated with local health-promoting measures.

## Background

Overweight and obesity are important health problems among children and adolescents in the Western countries [1-3]. A study conducted in Stockholm County, Sweden in 2003 showed that 20.5% of all boys were overweight, and 3.8% were obese. For girls, the corresponding prevalence estimates were 19.2% and 2.8% [4]. Place of residence has been shown to be significantly associated with body mass index (BMI) in late adolescence and adulthood [5,6]. Strong socio-economic gradients with higher prevalence of overweight and obese children and adolescents from disadvantaged groups have been reported in Sweden and Canada [7,8]. There are several mechanisms of the obesity epidemic, but physical activity and eating habits are strongly related to weight development at a population level. A recent study from Stockholm showed that 71% of 15-year-olds were physically active at a moderate or high level for 60 minutes per day or more [9]. It was also shown, however, that adolescents with a lower educated mother, those in overcrowded accommodation, and those with immigrant background were the most sedentary. It was recently reported that only about one-third of those aged 15 years and older in the European Union are physically active at the recommended levels [10], indicating that in many European countries adolescents may be less active than in Sweden. This also applies to children between the ages of 11 and 15 [11].

Adults can more or less make their own lifestyle choices, but children are left with parental decisions and socio-cultural family environment as well as structural factors related to schools, the local community, and society as a whole [10]. Interventions at the family level will depend on the families' ability to follow advice and make behavioural changes, and results are therefore likely to be related to social class and parental educational level. Interventions at school and/or municipal levels, however, provide good opportunities to set up structures that support physical activity and healthy eating habits reaching all children, regardless of socio-economic family position. Such structures can be either obesogenic, meaning that they prevent or hinder healthy behaviours, or leptogenic, meaning that they encourage healthy behaviours [12,13]. A theoretical framework based on obesogenic and leptogenic environments has been developed by Swinburn and colleagues. This framework is divided into the political environment, the physical environment, the economic environment, and the socio-cultural environment [12]. Examples related to physical activities are adjustments of infrastructure such as traffic-calming measures aiming to increase pedestrian and bicycle safety [14,15]. In a systematic review, van Sluijs, McMinn, and Griffin stated that interventions, including both school and family or community involvement, have a better potential to increase levels of physical activity among adolescents than interventions focusing only on one of these levels. [16]. Research has also shown that access to facilities such as parks and activity programmes and time spent outdoors are positively related with levels of physical activity among children [17]. The Guidelines for School Lunches, developed in Sweden by the Swedish National Food Administration, is an example of structural factors promoting healthy eating habits. Other examples of health-promoting factors are absence of soda machines and candy stores in and around schools and food policies in schools [18,19].

The factors described above are examples of environments supporting physical activity and healthy eating habits. Starting with the Ottawa conference in 1986, a new broader understanding of health promotion was adopted [20]. It was subsequently realised that changes at the societal level often is a more feasible and efficient way of facilitating lifestyle changes at a population level than interventions aimed at behavioural change at the individual level [21], and policy-making became an issue on the public health arena. The policy process is often described in several stages, *e.g*., problem identification, policy formulation, policy implementation, and policy evaluation [22]. According to this, structured public health work normally comprises policies, plans of action, implementation measures, and evaluations. Such structured work has been shown to be successful, *e.g*., in safety promotion [23].

In Sweden, the municipalities are accountable for the main part of the arenas where children and adolescents spend considerable amounts of their time, *e.g*., preschools and schools as well as local infrastructure such as the route to school, playgrounds, and leisure environments. The Swedish compulsory school comprises children aged six to 16. Swedish municipalities have, like municipalities in many other welfare states [24], unique conditions regarding self-government, democratic control, and tax equalisation that take into account age distribution, tax-paying capacity, and population density. This autonomy implies that while the national government legislates on the building, traffic, and school environments, it cannot prescribe exactly how the local governments shall put these laws into practice. Public authorities, such as the Swedish National Institute of Public Health, have developed public health objectives that also address healthy eating and physical activity. These objectives are not imperative, however, but guiding principles for the municipalities. In large municipalities, the local government is often decentralised to town districts. Thus, municipalities and town districts seem to be the proper arenas for structural health-promoting actions that aim to reach a large proportion of the children and adolescents.

In the light of these circumstances, the aims of our study were threefold: first, we wanted to investigate whether policies and plans of action at a central municipal level increased the number of measures carried out to promote physical activity and healthy eating habits among schoolchildren aged six to 16; second, we intended to investigate to which extent such measures were given priority in municipalities and town districts, *i.e*., whether physical activity and healthy eating habits among schoolchildren were judged to be important by local decision-makers; third, we wished to explore whether municipal demographic and socio-economic characteristics were associated with the amount of local measures carried out to promote physical activity and healthy eating habits among schoolchildren.

## Methods

In this cross-sectional study, questionnaires and public statistics were used to collect data from all municipalities (9,000 to 91,000 inhabitants) in Stockholm County. In addition, the town districts of the municipality of Stockholm, the largest municipality in Sweden (794,500 inhabitants), were included.

Indicators were developed and survey questions constructed in order to capture the work carried out by the municipalities at a structural level to enable children and adolescents to be physically active and to develop healthy eating habits. This was done by searching the literature and by consulting health-planning officers and other experts in the municipalities. We used the theoretical framework for obesogenic environments developed by Swinburn and colleagues, which is divided into the political environment (*e.g*., policies, plans of action and systematic follow-up at the central level), the physical environment (*e.g*., ability to walk and bike and public accessibility to sports facilities), the economic environment (*e.g*., free or subsidised entrance to sports facilities, subsidies to sports clubs, and free school lunches) and the socio-cultural environment (*e.g*., attitudes to health promotion among decision makers, public officials, and school headmasters) [12]. Attitudes to health promotion among municipal decision makers were supposed to be revealed through questions regarding how health-promoting measures were prioritised in the municipality. The Swinburn conceptual framework was used to construct and categorise the blocks of questions used in the questionnaires (Table 1). The first part of the questionnaire aimed to identify and characterise structured public health work (the political environment), and was built on three often-mentioned stages in the policy process [22]: policy formulation (are there any policies aiming to promote physical activity and/or healthy eating habits, and are there any plans of action aiming to promote physical activity and/or healthy eating habits?), policy implementation (are any measures of implementation taken to promote physical activity and/or healthy eating habits?), and policy evaluation (are systematic follow-ups of implemented measures performed?) [25-27]. The concepts policy, plan of action, and evaluation were defined in the questionnaire. In order to distinguish measures related to the physical, economic, and socio-cultural environments, questions based on previous research [28-39] were used (Table 1).

**Table 1:** Environmental perspectives (Swinburn *et al*. 1999) related to survey questions supported by previous research.

Environmental perspectives	Survey questions	References
Political environment	Are there any *policies *aiming to promote physical activity and/or healthy eating?	[25-27]
		
	Are there any *plans of action *aiming to promote physical activity and/or healthy eating?	
		
	Are there any *implementation measures *made to promote physical activity and/or healthy eating?	
		
	Are *systematic follow-ups *of implemented measures performed?	
		
	Are objectives, plans and evaluations regarding physical activity stated in the municipal school plan?	
		
	Are objectives, plans and evaluations regarding healthy eating stated in the municipal school plan?	

Economic environment	Are there any incentives provided in order to increase the use of sports centres among children?	[28,29]

Socio-cultural environment **(Attitudes to health promotion among municipal decision-makers were supposed to be revealed by questions regarding how health-promoting measures were prioritised in the municipality)**	Are there any measures taken in order to increase walking and biking to school and in general?	[30,31]
		
	Have any overhauls of walking and bike paths been performed in the last five years?	
		
	Have any overhauls of walking and bike paths to and from schools been performed in the last five years?	
		
	Have any overhauls of the traffic safety in the immediate vicinity of the schools been performed in the last five years?	
		
	Compared to the municipal road network, how prioritized are the bike paths regarding maintenance during wintertime?	
		
	Is there any public health officer or similar staff employed in the municipality?	
		
	Is there any diet head or diet coordinator employed in the municipality?	

Physical environment	Are up-to-date and weatherproof bike stands provided?	[32-39]
		
	Are bike paths maintained during wintertime?	
		
	Has a general speed limit of 30 km/h been implemented in housing areas?	
		
	Part of total bike paths separated from road traffic	
		
	Kilometers of biking paths in relation to municipal road network.	
		
	Kilometers of walking paths in relation to municipal road network.	

The questionnaires were sent by mail to all municipalities (N = 25) in Stockholm County in late 2005 and early 2006. Due to the large population size of the Stockholm municipality, the local political and administrative responsibilities have been delegated to town districts. Accordingly the questionnaires were also sent to all town districts in the Stockholm municipality (N = 18). Two written reminders and one final reminder by telephone were given. The response to each question was coded with the intention to reflect the level of measures. Question group scores were computed within each block of questions (the political, physical, economic, and socio-cultural environment). These question group scores were designed to mirror the measures taken within each block of questions. The measures were not weighed regarding quality of evidence or reach into the municipalities. Thus, all measures were given equal weight. Only fully appropriate responses to the questions were scored as if the municipality or town district provided significant activity, as explained in Table 2.

**Table 2:** Measures taken aiming to facilitate physical activity and healthy eating habits among school children in 23 municipalities and 17 town districts in Stockholm County

Environmental perspectives	Survey questions	Number of municipalities with significant measures taken	Number of town districts with significant measures taken
Political environment	Are there any policies aiming to promote physical activity and/or healthy eating? *Significant measures taken = Yes, AND the policy should be attached AND be politically adopted AND be of present interest AND contain clear and measurable aims*.	6	1
	
	Are there any plans of action aiming to promote physical activity and/or healthy eating? *Significant measures taken = Yes, AND the plan of action should be attached AND be politically adopted AND be of present interest AND contain clear guiding principles on how to reach the aims in the policy*.	3	2
	
	Are there any implementation measures made to promote physical activity and/or healthy eating? *Significant measures taken = Yes, and there is a responsible person*	1	2
	
	Are systematic follow-ups of implemented measures performed? *Significant measures taken = Yes, and there is a responsible person*	0	2
	
	Are objectives, plans, and evaluations regarding physical activity stated in the municipal school plan? *Significant measures taken = Yes, AND the school plan should be attached AND contain measurable aims AND follow-up intentions AND a responsible person*	8	2
	
	Are objectives, plans, and evaluations regarding healthy eating stated in the municipal school plan? *Significant measures taken = Yes, AND the school plan should be attached AND contain measurable aims AND follow-up intentions AND a responsible person*	8	2

Economic environment	Are there any incentives provided in order to increase the use of sports centres among children? *Significant measures taken = Yes, AND a sufficient example*	5	5

Socio-cultural environment	Are there any measures taken in order to increase walking and biking to school and in general? *Significant measures taken = Yes, measures are taken to increase both walking and biking to school*	14	11
	
	Have any overhauls of walking and bike paths been performed in the last five years? *Significant measures taken = Yes, all walking and bike paths*	9	4
	
	Have any overhauls of walking and bike paths to and from schools been performed in the last five years? *Significant measures taken = Yes, all walking and bike paths to and from schools*	4	1
	
	Have any overhauls of the traffic safety in the immediate vicinity of the schools been performed in the last five years? *Significant measures taken = Yes, a TOTAL overhaul*	5	2
	
	Compared to the municipal road network, how prioritised are the bike paths regarding maintenance during wintertime? *Significant measures taken = more important than the road network*	6	8
	
	Is there any public health officer or similar staff employed in the municipality? *Significant measures taken = Yes*	11	0
	
	Is there any diet head or diet coordinator responsible for nutritional quality of meals served in institutions in the municipality? *Significant measures taken = Yes*	18	7

Physical environment	Are up-to-date and weatherproof bike stands provided? *Significant measures taken = Yes, there are up-to-date bike stands at all schools AND most bike stands are weatherproof*	5	5
	
	Are bike paths maintained during winter time? *Significant measures taken = Yes, the whole bike path network*	13	10
	
	Has a general speed limit of 30 km/h been implemented in housing areas? *Significant measures taken = Yes, in all housing areas*	3	11
	
	Part of total bike paths separated from road traffic *Significant measures taken ≥ 90%*	11	
	
	Km of biking paths in relation to municipal road network. *Significant measures taken ≥ median (0.52)*	5	
	
	Km of walking paths in relation to municipal road network. *Significant measures taken ≥ median (0.41)*	7	

As a validity measure, policy documents and plans of action were gathered from the municipalities and town districts, and compared to the answers given in the questionnaires. The survey questions 'are there any policies aiming to promote physical activity and/or healthy eating among schoolchildren?' and 'are there any plans of action aiming to promote physical activity and/or healthy eating among schoolchildren?' were compared to the collected policy documents and plans of action and coded in the following manner: five criteria had to be fulfilled in order for these questions to be validated and coded as 'yes, there exists a policy/plan of action', namely: the response in the questionnaire should be 'yes'; the policy/plan of action should be attached; the attached policy should be politically adopted; the attached policy should be of contemporaneous relevance; and the attached policy should contain clear and measurable aims regarding physical activity and/or healthy eating habits among children and adolescents. Answers to question two and three were mostly found in the attached policy documents and plans of action, but also on the websites of the municipalities. Questions three to five in the validity control also constituted a means of checking the quality of the policy documents and plans of action. If a policy document was not approved in the municipal executive board or if there were no clear and measurable aims, the quality of the policy document was assessed to be low. Two researchers (first and second author) were independently involved in the quality check of all the policy documents and plans of action.

Information regarding municipal demographic and socio-economic characteristics (total population size, annual population growth, and proportion of adults with more than 12 years of education) were gathered from public statistics [40,41].

Statistical analysis comprise Spearman rank correlations [42] estimated by the SAS software package. Associations between different question groups as well as between question groups and demographic and socio-economic characteristics were assessed. Data from municipalities and town districts were analysed both separately and together.

## Results

Twenty-three of 25 municipalities and 17 of 18 town districts completed the questionnaire. Twelve municipalities and five town districts attached policy documents, and five municipalities and six town districts attached plans of action. Of the attached documents, only seven policy documents and three plans of action from the municipalities, and five policy documents and one plan of action from the town districts were approved. Not a single municipality and only one town district could present the whole chain of structured public health work, such as valid policy documents, valid plans of action for implementation, and evaluation measures.

The structural variables comprise municipal measures enabling children and adolescents to be physically active and to develop healthy eating habits. The variables were divided into four question groups as described above – political environment, physical environment, economic environment, and socio-cultural environment. No significant associations were found between the four question groups (Table 3). The political environment thus does not seem to be associated with measures that are implemented in municipalities and town districts in order to promote physical activity and healthy eating habits among schoolchildren. A correlation of borderline statistical significance (r_s _= 0.31, p = 0.055) appeared between socio-cultural environment and physical environment. This could imply that in municipalities and town districts where public officials and politicians have a positive attitude to health promotion more and better measures are taken to enhance the physical environment (*e.g*., maintenance of bike paths). Although Swedish municipalities often invest in various health-promoting measures, especially aimed at children and adolescents, the measures asked for in this study do not seem to be given high priority by local decision-makers.

**Table 3:** Spearman rank correlations between questions groups related to structures in society aiming to facilitate physical activity and healthy eating habits among school children in 23 municipalities and 17 town districts in Stockholm County.

	Political environment	Physical environment	Economic environment
***Municipalities and town districts***			

**Physical environment**	-0.003 (0.985)		

**Economic environment**	-0.16 (0.319)	-0.18 (0.254)	

**Socio-cultural environment**	0.12 (0.444)	*0.31 (0.055)*	0.10 (0.529)

***Municipalities only***			

**Physical environment**	0.03 (0.882)		

**Economic environment**	-0.25 (0.241)	-0.32 (0.132)	

**Socio-cultural environment**	-0.05 (0.829)	0.25 (0.249)	0.35 (0.101)

***Town districts only***			

**Physical environment**	-0.18 (0.486)		

**Economic environment**	0.00	0.03 (0.917)	

**Socio-cultural environment**	0.07 (0.794)	0.40 (0.107)	-0.17 (0.500)

p < 0.05 significant

p < 0.10 tendency

Demographic and socio-economic characteristics at the municipal level (total population size, annual population growth, and proportion of adults with more than 12 years of education) were analysed in relation to the structural variables (Table 4). The results for municipalities and town districts are presented both separately and jointly. Town districts with a higher total population size offered more measures aiming to promote physical activity and healthy eating habits among schoolchildren (r_s _= 0.53, p = 0.030) than municipalities and town districts with lower total population size. Investments related to the physical environment were higher in those town districts where a higher proportion of the adult population had attained more than 12 years of education (r_s _= 0.74, p = 0.006). For the municipalities an inverse association (r_s _= -0.41, p = 0.054) was seen between annual population growth and the number of measures taken with the aim to promote physical activity and healthy eating habits among schoolchildren.

**Table 4:** Spearman rank correlations between questions groups related to structures aiming to facilitate physical activity and healthy eating habits and selected socio-economic and demographic characteristics of 23 municipalities and 17 town districts in Stockholm County.

Questions groups related to structures aiming to facilitate physical activity and healthy eating habits	Total population size	Annual population growth	Adults with >12 years of education
***Municipalities and town districts***			

**Political environment**	0.25 (0.111)	-0.18 (0.275)	0.20 (0.219)

**Physical environment**	0.26 (0.106)	-0.16 (0.321)	*0.36 (0.022)*

**Economic environment**	-0.03 (0.854)	-0.01 (0.927)	-0.09 (0.580)

**Socio-cultural environment**	0.27 (0.097)	0.007 (0.965)	-0.12 (0.474)

**Total score**	*0.36 (0.022)*	-0.09 (0.580)	0.05 (0.753)

***Municipalities only***			

**Political environment**	0.18 (0.408)	-0.28 (0.201)	-0.18 (0.407)

**Physical environment**	0.15 (0.486)	-0.39 (0.068)	0.20 (0.350)

**Economic environment**	0.09 (0.665)	0.34 (0.110)	-0.08 (0.718)

**Socio-cultural environment**	0.22 (0.308)	-0.27 (0.211)	-0.33 (0.120)

**Total score**	0.25 (0.255)	*-0.41 (0.054)*	-0.13 (0.543)

***Town districts only***			

**Political environment**	0.44 (0.076)	-0.09 (0.736)	0.00

**Physical environment**	0.46 (0.062)	0.12 (0.637)	*0.74 (0.006)*

**Economic environment**	-0.21 (0.417)	-0.32 (0.206)	-0.24 (0.359)

**Socio-cultural environment**	0.35 (0.170)	0.20 (0.430)	0.30 (0.237)

**Total score**	*0.53 (0.030)*	0.23 (0.381)	*0.56 (0.018)*

p < 0.05 significant

p < 0.10 tendency

A finding not be overlooked was the lack of agreement between what was reported in the questionnaires as local policies and plans of action and the relatively pointless documents actually observed by the investigators when scrutinising and comparing the responses with the attached documents. Out of a total of 28 attached policy documents and plans of action, only 16 were of a high enough quality to be approved.

## Discussion

The main finding of this study was that policy documents and plans of action aiming to promote physical activity and healthy eating habits among schoolchildren in municipalities and town districts did not seem to be associated with ongoing health-promoting measures. By contrast, our results indicate that demographic and socio-economic characteristics at the municipal level were associated with the amount and level of measures. In town districts with a high total population size, more health-promoting actions were reported. This was also the case in municipalities and town districts with a high proportion of adults with more than 12 years of education. In municipalities with a high annual population growth, less action to promote healthy eating and physical activity patterns was seen than in municipalities with a lower annual population growth.

The structured public health work in the municipalities and town districts in the Stockholm County seemed to have serious limitations regarding actions aiming to enable schoolchildren to be physically active and develop healthy eating habits. Policies of sufficient quality were rare, and plans of action even more uncommon. Implementation measures and evaluations of the complete chain of structured public health work, from policy, plan of action, to implementation, hardly existed anywhere. Furthermore, when the answers in the questionnaires were compared to attached documents, it became obvious that what municipalities and town districts labelled policies and plans of action aiming to promote physical activity and healthy eating habits could in fact not be considered as such. A variety of shortcomings appeared, *e.g*., the policies and plans of action were not politically adopted, not currently valid, or did not contain clear and measurable aims. Some of these documents might therefore be difficult to implement and perhaps even more difficult to evaluate. There are several reasons for the discrepancy between what was reported in the questionnaires and what was stated in the gathered documents. The municipalities and town districts may have wished to exaggerate their public health work when responding to the questionnaire or they may not have been fully aware of the weaknesses of their policies and plans of action. Because no municipality and only two town districts have evaluated their policies and plans of action, it must have been difficult to realise their limitations and potential lack of impact. The overall lack of evaluation is noteworthy. Another possible reason for the discrepancy between what was reported in the questionnaires and what was stated in the gathered documents is that the 'wrong' people may have completed the questionnaires. No specific person in the municipalities was addressed. Instead it was explained in the cover letter which topics the questionnaire concerned and suggested which professions might be the appropriate respondents.

It is somewhat surprising that there were no clear-cut associations between structured health-promoting work according to answers related to the political environment and answers related to the physical, economic, and socio-cultural environments in the municipalities and town districts. Could this lack of association be a consequence of limitations of the questionnaire intended to measure policies, plans of action, and evaluation? The authors do not believe so, as a thorough validity check was performed by comparing questionnaire responses with policy documents and plans of action gathered, and only responses that could be validated against attached documents were approved. Instead, it is possible that structural measures aiming to positively influence physical activity and eating habits among schoolchildren were given rather low priority in the municipalities and town districts. The goals in the policy documents were mostly rather vague and not easy to turn into specific objectives that could be evaluated later on. Means of reaching the goals in the policies were seldom clearly specified in the plans of action. Therefore, it may be considered whether the rather low quality of the policies and plans of action might hamper local actions and measures. A final interpretation of the lack of association between structured health-promoting work related to the political environment and measures related to the physical, economic, and socio-cultural environments is the well-known difficulties of implementing new methods in everyday practice in general [43,44]. The strikingly negative response (see Table 2) to the question 'are there any implementation measures taken to promote physical activity and/or healthy eating habits?' makes this interpretation plausible.

Structured public health work, comprising policies, plans of action, implementation measures, and evaluations have convincingly been shown to be successful in safety promotion [23]. So how do measures aiming to promote physical activity and healthy eating habits among Swedish schoolchildren differ from measures on the international arena of safety promotion? Within the safety promotion area, the importance of the whole chain (policies, plans of action, implementation measures, and evaluation) of structured public health work has been emphasised, for example, by systematic injury registration. In the present study, only the first part of the process (policies and plans of action) is discernable, and, in fact, shows very low quality. Thus, the important components of implementation measures and evaluation are missing. The linear approach to the policy process has been discussed among policy researchers [22,45]. It is argued that the policy process is more complex and non-linear. The linear approach is helpful in gathering and structuring data, although we must be careful with implications based on a presumed linear policy process.

Regarding the influence of municipal demographic and socio-economic characteristics, similar results have been reported from previous studies. Guldbrandsson and Bremberg showed that growing municipalities reported fewer safety-promoting measures [46] and fewer measures aiming to promote mental health among preschool children [47] than municipalities with a stable population size. On the other hand, in these studies the proportion of the adult population with more than 12 years of education was not associated with the amount of health-promoting actions, *i.e*., the intentions to promote young people's health appeared to be similar in municipalities with higher and lower percentage of well-educated people. This goes against the results of the present study that show a clear-cut positive association between the proportion of well-educated adults and measures aiming to improve the physical environment in both municipalities and town districts. Positive associations probably depend on higher tax-paying capacity and higher demands for municipal services among well-educated people. The discrepancy between the previous studies and the present study might be explained by changes in the Swedish national system for equalising municipal resources that went into effect on 1 January 2005.

## Conclusion

Policy documents and plans of action aiming to promote physical activity and healthy eating habits among schoolchildren in municipalities and town districts in Stockholm, Sweden did not seem to be associated with ongoing health-promoting measures at the local level. Demographic and socio-economic characteristics, however, seemed to be associated with such measures. There was no agreement between what was reported in the questionnaires concerning the existence of local policies and plans of action and what was observed by the investigators when scrutinising requested documents attached to the questionnaires. Researchers and policy makers should thus be aware of potential discrepancies between what is declared in policies and plans, often worded in general terms, and what is actually done at the local municipal level. High-quality local policies and plans of action must be developed, implemented, and evaluated to assess whether the low impact revealed in the present study is the consequence of poor-quality documents. Local implementation and evaluation efforts must be strengthened.

## Competing interests

The authors declare that they have no competing interests.

## Authors' contributions

All authors contributed significantly to the development of the research questions, plan for analyses, interpretation of results, and drafting the paper. KMW was mainly responsible for developing the questionnaire, data collection, and statistical analyses. FR developed the research proposal and was the holder of a grant from the Public Health Committee, Stockholm County Council. All authors contributed significantly to the final version.
